# Scalable topographies to support proliferation and Oct4 expression by human induced pluripotent stem cells

**DOI:** 10.1038/srep18948

**Published:** 2016-01-13

**Authors:** Andreas Reimer, Aliaksei Vasilevich, Frits Hulshof, Priyalakshmi Viswanathan, Clemens A. van Blitterswijk, Jan de Boer, Fiona M. Watt

**Affiliations:** 1 Centre for Stem Cells and Regenerative Medicine, King’s College London, 28th Floor, Tower Wing, Guy’s Hospital, Great Maze Pond, London SE1 9RT, United Kingdom; 2 MIRA Institute for Biomedical Technology and Technical Medicine, Enschede, The Netherlands; 3 Materiomics b.v., Maastricht, The Netherlands; 4 Merln Institute for Technology-inspired Regenerative Medicine, Maastricht University, Maastricht, The Netherlands

## Abstract

It is well established that topographical features modulate cell behaviour, including cell morphology, proliferation and differentiation. To define the effects of topography on human induced pluripotent stem cells (iPSC), we plated cells on a topographical library containing over 1000 different features in medium lacking animal products (xeno-free). Using high content imaging, we determined the effect of each topography on cell proliferation and expression of the pluripotency marker Oct4 24 h after seeding. Features that maintained Oct4 expression also supported proliferation and cell-cell adhesion at 24 h, and by 4 days colonies of Oct4-positive, Sox2-positive cells had formed. Computational analysis revealed that small feature size was the most important determinant of pluripotency, followed by high wave number and high feature density. Using this information we correctly predicted whether any given topography within our library would support the pluripotent state at 24 h. This approach not only facilitates the design of substrates for optimal human iPSC expansion, but also, potentially, identification of topographies with other desirable characteristics, such as promoting differentiation.

Human induced pluripotent stem cells (iPSC) offer the exciting prospect of treating diseases that are currently intractable^1^. To achieve that goal, efficient expansion of cells in the pluripotent state and in the absence of animal products (xeno-free conditions) is desirable. Although xeno-free media such as Essential 8 (E8) have been developed^2^, survival, growth and self-renewal of iPSC require cell attachment to an adhesive substrate, which is typically presented in the form of extracellular matrix (ECM) components such as vitronectin, Geltrex or laminin-511^3^,^4^,^5^. Replacing ECM proteins with a completely artificial substrate not only avoids exposing cells to animal proteins, but also increases reproducibility and potentially reduces costs. Some progress in that direction has already been made, through the development of synthetic polymer coatings^6^ or acrylate surfaces incorporating cell adhesive peptides^7^. However, there is a need for better high throughput approaches to substrate design.

Although cell culture surfaces are typically flat, there is good evidence that cells also respond to topographical features at the nano- and micro-scale^8^. Surfaces that incorporate topographical features can support the growth and differentiation of mouse and human pluripotent stem cells in serum-containing medium^9^,^10^,^11^,^12^. By assaying cell behaviour quantitatively on a library of different topographical features^13^ and applying computational analysis it is possible to predict cellular responses to topographical features prior to experimental analysis^14^. With these considerations in mind, we plated human iPSC in xeno-free medium without added ECM proteins on a library of over 1000 topographies to identify, in an unbiased manner, topographical features that maintain pluripotency.

## Results

### Screening the topographical library

**Figure 1:**
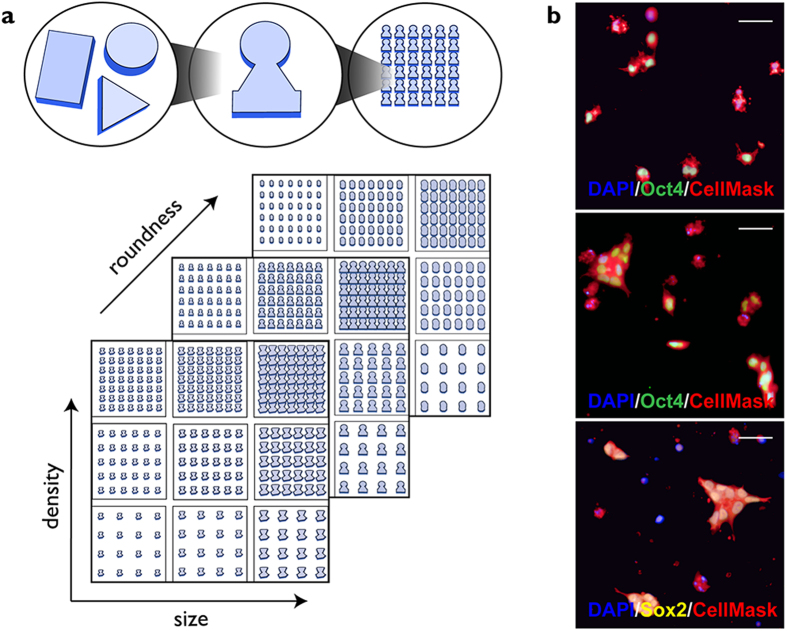
Design of TopoUnits and iPSC screen. (**a**) Schematic of the topography library. Top: combining circular, triangular and rectangular primitives into specific features that are repeated within a single TopoUnit. Bottom: differences in feature size, density and roundness between individual TopoUnits arrayed in a TopoChip. (**b**) Individual TopoUnits seeded for 4 h (top) or 24 h (middle, bottom) then labelled for Oct4 (green) or Sox2 (yellow), CellMask (red) and DAPI (blue). Scale bars: 50 μm.

We plated cells on the previously described TopoChip library, which comprises 2,176 distinct surface topographies in duplicate on a 2 × 2 cm^2^ TopoChip platform^13^. Each topography is arrayed in an area of 290 × 290 μm^2^, referred to as one TopoUnit. The topographies are based on combinations of circles, squares and rectangles with a feature height of 10 μm and vary in attributes such as feature size, density and roundness^13^ (Fig. 1a). Fabrication of the TopoChip platform utilizes hot embossing of standard tissue culture polystyrene, reducing the cost of manufacture and enabling future large-scale culture on selected topographies (Zhao *et al.* submitted).

To best evaluate the ability of human iPSC to grow as single cells, topographies were seeded at low density (100 cells/mm^2^, corresponding to approximately 12 cells per TopoUnit) in E8 medium. The medium was supplemented with Rho-associated kinase (ROCK) inhibitor, which prevents dissociation-associated apoptosis^12^. An assay time of 24 hours was chosen to capture the initial cellular responses to the topographies. 5-ethynyl-2′-deoxyuridine (EdU) was added for the final 30 min to label S phase cells^15^. Following fixation, cells were labelled with antibodies to Oct4 as a marker of pluripotency^16^. The plasma membrane dye CellMask was used to distinguish individual cells versus groups of cells. DAPI was added as a DNA label to identify individual nuclei. Four hours after seeding, the majority of attached cells were single cells (Fig. 1b). After 24 hours, most cells were in clusters, which formed by a combination of cell proliferation and migration (Fig. 1b). In addition to expressing Oct4, undifferentiated iPSC expressed Sox2^16^ (Fig. 1b).

### Quantitation of EdU and Oct4 labelling

**Figure 2:**
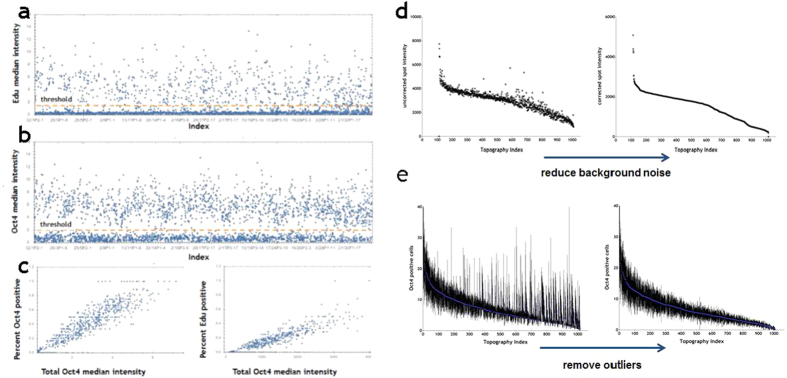
Quantitation of EdU+ and Oct4+ cells. (**a,b**) Fluorescence intensity of individual cells is shown on y axes; x axes show individual TopoUnits. Position of thresholds for scoring individual cells as EdU+ (**a**) or Oct4+ (**b**) is shown. **(c)** Relationship between median Oct4 staining intensity and % Oct4+ or EdU+ cells on individual TopoUnits. R-values 0.1149 and 0.1188, respectively. (**d,e**) Effects of reducing background noise and outliers on ranking topographies according to the number of Oct4+ cells per TopoUnit. Signal was increased by removal of background noise (**d**) and outliers were defined as 2.5 SD away from mean (**e**).

The nuclear fluorescence intensity of all individual cells labelled with EdU or Oct4 on each TopoUnit was measured by high content imaging (Fig. 2a,b). To score individual cells as positive or negative, thresholds were set for each label (Fig. 2a,b). There was a linear relationship between the total Oct4 median intensity per TopoUnit and % Oct4+ cells (Fig. 2c). This was also observed when Oct4 median intensity per TopoUnit was plotted against % EdU+ cells (Fig. 2c). We then analysed 1000 topographies in detail, discarding 18 as unreadable due to defects in the manufacturing process. The effects of reducing background noise (Fig. 2d) and outliers (Fig. 2e) on ranking topographies according to the number of Oct4+ cells per TopoUnit are shown Fig. 2.

**Figure 3:**
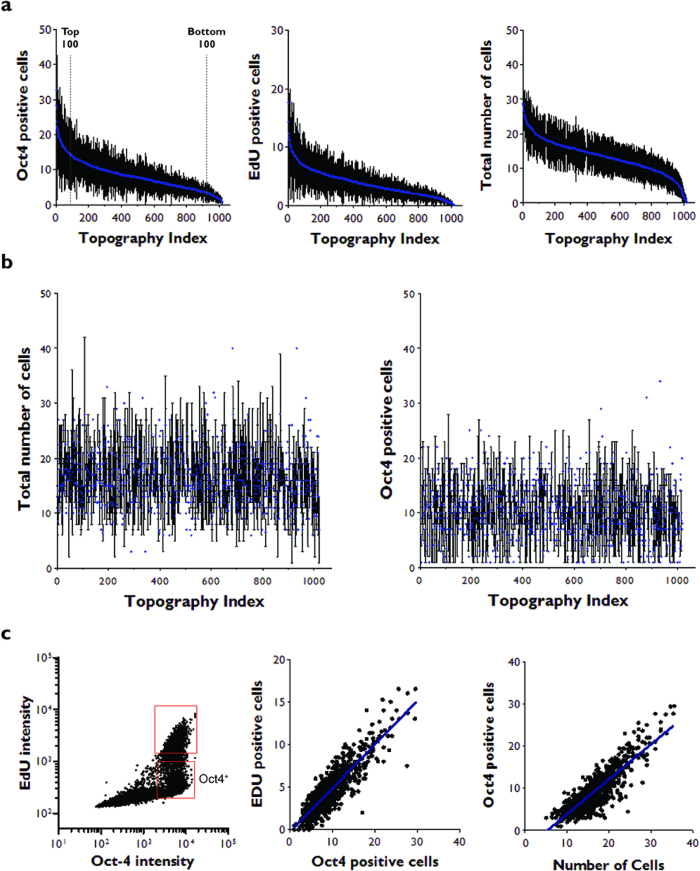
Human iPSC proliferation and Oct4 expression on distinct topographies. (**a**) Topographies were ranked according to the total number of Oct4+ (left) or EdU+ cells (middle) or the total number of attached cells (right) at 24 h. Cutoffs for the 100 topographies with the highest and lowest number of Oct4 positive cells are shown. (**b**) Total number of cells (left) and number of Oct4+ cells (right) at 4 h. Topographies were ranked according to total number of Oct4+ cells at 24 h (**a**). (**c**) Fluorescence intensity thresholds were set for Oct4 (Oct4^+^) and EdU (EdU^+^) cells (Fig. 2) and the number of positive cells for each marker was determined. Left hand panel shows all the cells (individual dots) on a single TopoChip. Top gate contains EdU+ Oct4+ cells; bottom gate contains Oct4+ EdU− cells. Middle and right hand panels show all topographies (TopoUnits) on a single TopoChip. The R^2^ coefficient of determination values was calculated for positive correlations between Oct4+ and EdU+ cells (middle panel; R^2^ = 0.78), or Oct4+ cells and total cell number (right hand panel; R^2^ = 0.74).

Thereafter, topographies were ranked according to the total number of cells per TopoUnit, the number of Oct4+ cells, and the number of EdU+ cells at 24 h (Fig. 3a; n = 6 TopoUnits). The total number of cells and the number of Oct4+ cells per TopoUnit at 4 h did not correlate with the TopoUnit rankings at 24 h (Fig. 3b), indicating that cell properties at 24 h did not simply reflect the ability of TopoUnits to support initial adhesion of Oct4+ cells.

By pooling the measurements for all cells on one TopoChip at 24 h, we established that essentially all EdU+ cells were also Oct4+, but approximately 30% of Oct4+ cells were EdU− (Fig. 3c). By plotting the total number of cells and the number of EdU+ and Oct4+ cells per topography at 24 h, we observed linear correlations between the numbers of Oct4+ cells, EdU+ cells and total cells (Fig. 3c). Thus at 24 h TopoUnits with the greatest number of cells also had the greatest number of EdU+ cells and Oct4+ cells, indicating that they supported self-renewal and prevented differentiation of iPSC at this time point.

### Identifying topographical features that promote or decrease human iPSC proliferation and Oct4 expression

**Figure 4:**
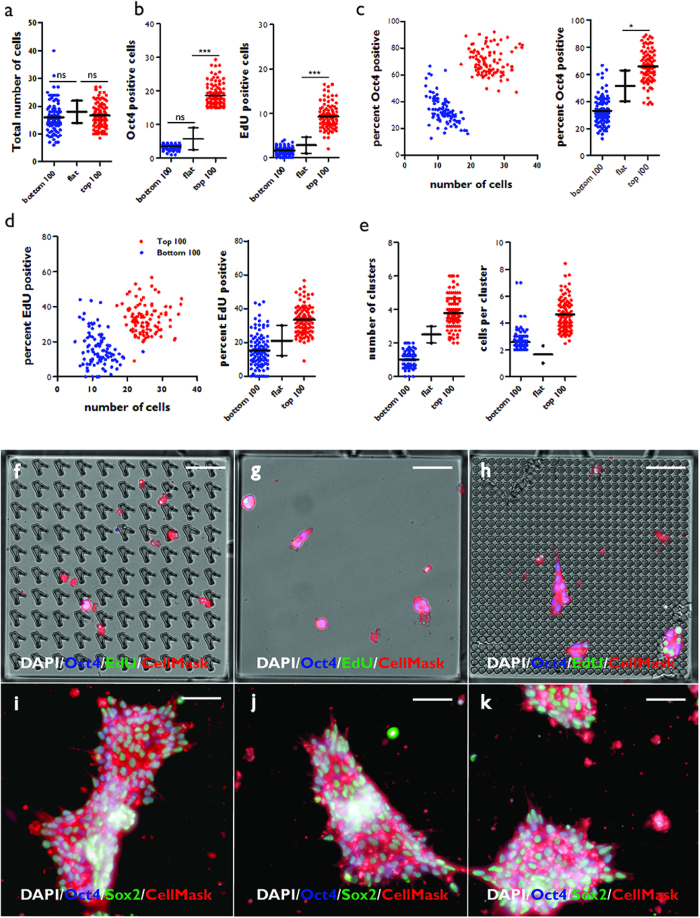
Comparison of cell behaviour on Top and Bottom 100 topographies. (**a–e**) Two hit categories were established by ranking topographies, as shown in Fig. 3a: the 100 topographies (TopoUnits) with the highest (Top 100) and lowest (Bottom 100) number of Oct4+ cells at 24 h. For each category the total number of attached cells at 4 h (**a**) and the number of Oct4+ and EdU+ cells per topography at 24 h (**b**) were quantified and compared with a flat topography (polystyrene). % Oct4+ (**c**) and % EdU+ (**d**) cells were also plotted against total number of adherent cells. (**e**) The number of clusters and cells per cluster are shown for each category. *p < 0.05; **p < 0.01; ***p < 0.001; ns: not significant. 25% confidence intervals are shown. (**f–h**) Individual TopoUnits representing one of the bottom (**f**) or top (**h**) 100 topographies are shown, with flat polystyrene (**g**) for comparison. (**i–k**) Colony formation at 4 days on topographies that supported EdU+ and Oct4+ cells at 24 h. Scale bars: 55 μm.

In order to discover how specific topographies elicited specific responses, the 100 topographies ranked top or bottom on the basis of number of Oct4+ cells at 24 h (Fig. 3a) were compared with each other and also with control TopoUnits that had a flat surface (polystyrene) (Fig. 4a–e). The 100 topographies with the highest number of Oct4+ cells (Top 100) at 24 h and the 100 topographies with the fewest Oct4+ cells at 24 h (Bottom 100) did not differ significantly from flat polystyrene when total cell number was scored at 4 h (Fig. 4a).

At 24 h, the Top 100 topographies had approximately three-fold more Oct4+ and EdU+ cells compared to flat polystyrene (Fig. 4b). At 24 h topographies of the bottom 100 hits (Bottom 100) had a similar number of Oct4+ cells and EdU+ cells to flat polystyrene (Fig. 4b). The percentage of Oct4+ and EdU+ cells was also significantly higher in Top 100 compared to Bottom 100 and polystyrene substrates (Fig. 4c,d). Further analysis revealed that the Top 100 topographies supported formation of more cell clusters and a greater number of cells per cluster compared to the Bottom 100 topographies and flat polystyrene (Fig. 4e). Representative images of each type of topography are shown in Fig. 4f–h.

When cultures were maintained for a total of 4 days, Top 100 topographies supported formation of extensive colonies of undifferentiated iPSC that expressed Oct4 and Sox2 (Fig. 4i–k). However, at later times colonies overgrew the boundaries of the TopoUnits, precluding further analysis (data not shown).

### Using computational tools to predict the topographical features that are most supportive of iPSC proliferation and Oct4 expression

Because the topographies were designed from a combination of multiple parameters we used an algorithm that allows the identification and quantification of topographic cues using Classification and Regression Trees (CART)^17^,^18^ in order to define the cues that support self-renewal of human iPSC at 24 h. Characteristics evaluated by this method include the numbers of primitives (circles, triangles and lines [rectangles]) used to make each repeating feature (‘building block’ including surrounding space) in a TopoUnit, the area of each primitive, circle diameter, length of the shortest side of a triangle, and line length (Fig. 1a). We also factored in overall feature size (FeatSize: the size of the bounding square for the feature [10, 20 or 28 μm]) and wave number [WN]. Wave number represents the fraction of the total energy in the signal in sinusoids and is computed after applying discrete Fourier transformation to the image of a single Feature^14^.

**Figure 5:**
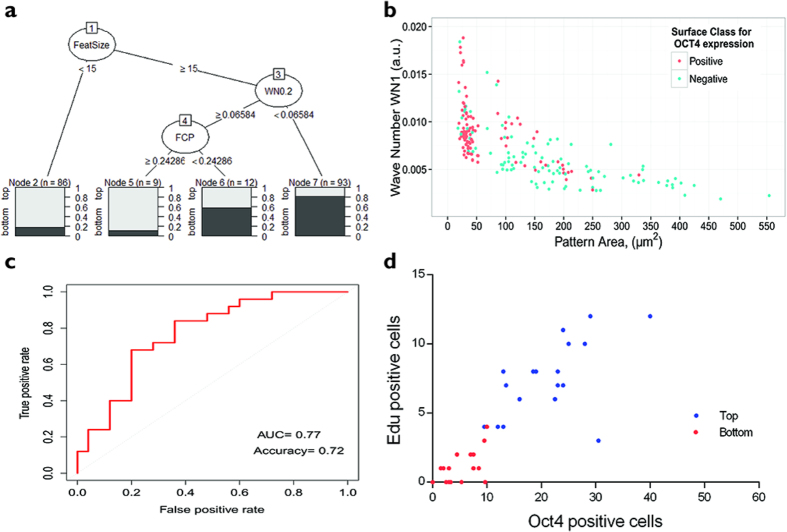
Identification of topography parameters that maintain proliferation and Oct4 expression. (**a**) Regression tree showing sorting of topographies. After each node, the fraction of Positive (top 100 for Oct4+ cells) versus Negative (bottom 100 for Oct4+ cells) topographies is shown. **(b)** Scatter plot showing distribution of pattern area and wave number parameters for topographies with highest (red; top hits) and lowest (blue; bottom hits) number of Oct4+ cells. **(c**) ROC plot showing prediction performance for logistic regression model. (**d**) 30 TopoUnits that had not been analysed previously were predicted to promote Oct4 expression and EdU labelling (‘top hits’; blue) and a further 30 were predicted to be unable to support those characteristics (‘bottom hits’; red). Of those TopoUnits, 19 in the top category and 15 in the bottom category were analysed and confirmed experimentally.

Using a regression tree of all hits from the Top 100 and Bottom 100 TopoUnits we found that small feature size was the most important determinant of pluripotency, followed by high wavenumber and high feature density (FCP) (Fig. 5a,b). More than 80% of the topographies in the Top 100 had a pattern area of less than 60 μm^2^. Pattern area was calculated by multiplying the area of each feature and the fraction of the feature covered by primitives (FCP). More than 80% of the Bottom 100 hits in the corresponding classification node had a wave number (WN0.2) smaller than 0.065 a.u. (i.e. small features more frequent) (Fig. 5a). If pattern area was greater than 60 μm^2^ and wavenumber (WN1) was less than 0.005 a.u., the majority of surfaces were not supportive of Oct4 expression (Fig. 5b).

We next employed a logistic regression direct probability model to identify further topographies that were predicted to support pluripotency at 24 h and topographies that were not. Logistic regression enables outcomes to be predicted on the basis of binary classification. The model we derived had an accuracy of 72%; the area under the curve for the Receiver Operating Characteristic (ROC) plot was 0.77, where randomly selected topographies are predicted to have an area under the curve value of 0.5 and predictor with 100% accuracy equal to 1 (Fig. 5c) . Of the 30 topographies predicted to support pluripotency, 24 were imaged and 19 proved to be top hits experimentally (Fig. 5d). Of the 30 topographies predicted not to support Oct4 expression, 25 were imaged and 15 proved to be bottom hits experimentally (Fig. 5d).

## Discussion

Topographical features have previously been shown to elicit responses in a variety of somatic and pluripotent cells^8^,^13^. Topography influences both generic aspects of cell behaviour, such as adhesion, cytoskeletal organisation and migration, and cell type-specific properties, such as selection of a specific differentiated lineage. The identification of signalling pathways that integrate different extrinsic stimuli to achieve specific outcomes is of considerable interest^19^. In the case of topographies, the simple parameter of cell size^20^ can potentially have a major impact on cellular responses, as a larger cell will have a different interface with a specific feature than a small cell, which will in turn affect cell spreading and cytoskeletal organisation.

We have demonstrated that human iPSC respond to the topographical features of the culture substrate and have defined features that support or decrease proliferation and Oct4 expression at 24 h, independent of the number of cells that attached at 4 h. Optimal topographies were able to support extensive colony formation and maintain Oct4 and Sox2 expression at 4 days. We conclude that by combining a topographical library with high content imaging and computational analysis, it is possible to design polystyrene substrates that support short-term maintenance of pluripotency in xeno-free medium in the absence of exogenous ECM. Our findings are not only of practical importance for future clinical applications of human iPSC but also raise interesting questions about how cells ‘read’ the physical constraints of their environment to select different fates.

## Methods

### (Poly)styrene topography fabrication

The TopoChip was designed by randomly selecting topographies from a vast in-silico library and fabricating each topography in a 66 by 66 array in a 300 × 300 μm micro-well to form a TopoUnit. The TopoUnits were then fabricated on a 2 × 2 cm^2^ chip as previously described, each chip containing a duplicate TopoUnit for each topography^13^.

(Poly)styrene (PS) TopoChips were prepared by hot embossing a PS film (Goodfellow) (Zhao *et al.* submitted). In summary, the inverse structure of the topographies was produced in silicon by standard photo lithography and deep reactive etching. The silicon mould was used to make a positive mould in poly(dimethylsiloxane) (PDMS). The PDMS mould was required to create a second negative mould in OrmoStamp hybrid polymer (micro resist technology Gmbh), which serves as the mould for hot embossing the PS films. Fabrication of the OrmoStamp mould circumvented the problem that demoulding PS from silicon often leads to destruction of either the mould or the Topochips.

### Cell culture

Human iPS cells (hSPC clone 2; kind gift of Prof. Jack Price, King’s College London) were generated from scalp hair keratinocytes using a polycistronic lentiviral construct^21^, maintained in Essential-8 (E8) medium on Geltrex (Invitrogen) and passaged twice weekly. When the cells were 70% confluent, and before colonies had begun to merge, cells were washed twice with HBBS, treated with EDTA for 5 minutes at 37 °C and removed from the dish by gentle pipetting. Cell clumps were transferred to fresh Geltrex-coated 6-well plates at a splitting ratio of 1:6 or 1:12, depending on the growth rate.

To harvest cells for plating on TopoChips, Accutase (Invitrogen) was added to cells two days after plating and the dish was incubated at 37 °C for 5–8 minutes. When the cells began to separate and round up, pre-warmed E8 medium containing 10 μM Rho- associated protein kinase (ROCK) inhibitor (Sigma-Aldrich) was added and cells were removed from culture wells by gentle pipetting to form a single cell suspension. Cells were then transferred into a 15 ml tube, centrifuged at 290 × g, aspirated and resuspended in E8 containing 10 μM ROCK inhibitor using a 1 ml pipette tip. Cells were counted using a Neubauer Chamber and the cell concentration was adjusted to 30–40 × 10^3 ^cells/ml. 4 ml cell suspension was added to each well containing a Topochip. To allow even cell distribution, plates were left at room temperature for 45 minutes until the cells were attached. Plates containing cells on TopoChips were then transferred to an incubator and cultured at 37 °C in 5% CO_2_.

### Immunohistochemistry and high content screening

Before fixation, EdU (Molecular Probes) was added to the medium for 30 minutes to detect proliferating cells. Half the medium was removed and replaced with 8% PFA in order to fix the cells without the risk of cell detachment. After washing twice with PBS, cells were labelled with anti-Oct4 antibody (Abcam), EdU (Molecular Probes), DAPI and cell mask (Invitrogen). In some experiments cells were also labelled with anti-Sox2 (R&D Systems). Each TopoChip was then mounted in Prolong antifade reagent (Life Technologies). An Operetta High Content Imaging System (PerkinElmer) was used to screen 1056 TopoUnits and standard algorithms were applied to quantify molecular markers at single cell resolution.

### Statistical analysis

To classify topographies that had a positive or negative effect on maintenance of pluripotency we used classification trees algorithms from the “rpart” package^22^ implemented in R ver. 3.1.2^23^. Before training the model all highly correlated features with r^2^ more than 0.75 were eliminated from further analysis. For creating the model we used 75% of the TopoUnits and the accuracy of the model was accessed on the remaining 25%. Models were trained with 10 fold cross validation in the “caret” package^24^. The classification tree was visualized using the “party” package^25^. The statistical techniques for classifying topographies that had a positive or negative effect on maintenance of pluripotency have been described previously^14^.

## Additional Information

**How to cite this article**: Reimer, A. *et al.* Scalable topographies to support proliferation and Oct4 expression by human induced pluripotent stem cells. *Sci. Rep.* **6**, 18948; doi: 10.1038/srep18948 (2016).
